# The Recombinant Protein EphB4-Fc Changes the Ti Particle-Mediated Imbalance of OPG/RANKL via EphrinB2/EphB4 Signaling Pathway and Inhibits the Release of Proinflammatory Factors In Vivo

**DOI:** 10.1155/2020/1404915

**Published:** 2020-06-05

**Authors:** Yu-Wei Ge, Kai Feng, Xiao-Liang Liu, Hong-Fang Chen, Zhen-Yu Sun, Cai-Feng Wang, Zhi-Qing Liu, Hao-Wei Wang, Jing-Wei Zhang, De-Gang Yu, Yuan-Qing Mao

**Affiliations:** ^1^Department of Orthopedic Surgery, Shanghai Ninth People's Hospital, Shanghai Jiao Tong University School of Medicine, Shanghai 200011, China; ^2^Department of Orthopedic Surgery, Shanghai Jiao Tong University Affiliated Sixth People's Hospital, Shanghai, China; ^3^Second Dental Clinic, Department of Oral Implantology, Ninth People's Hospital, College of Stomatology, Shanghai Jiao Tong University, School of Medicine, National Clinical Research Center for Oral Disease, Shanghai Key Laboratory of Stomatology & Shanghai Research Institute of Stomatology, Shanghai, China

## Abstract

Aseptic loosening caused by wear particles is one of the common complications after total hip arthroplasty. We investigated the effect of the recombinant protein ephB4-Fc (erythropoietin-producing human hepatocellular receptor 4) on wear particle-mediated inflammatory response. In vitro, ephrinB2 expression was analyzed using siRNA-NFATc1 (nuclear factor of activated T-cells 1) and siRNA-c-Fos. Additionally, we used Tartrate-resistant acid phosphatase (TRAP) staining, bone pit resorption, Enzyme-linked immunosorbent assay (ELISA), as well as ephrinB2 overexpression and knockdown experiments to verify the effect of ephB4-Fc on osteoclast differentiation and function. In vivo, a mouse skull model was constructed to test whether the ephB4-Fc inhibits osteolysis and inhibits inflammation by micro-CT, H&E staining, immunohistochemistry, and immunofluorescence. The gene expression of ephrinB2 was regulated by c-Fos/NFATc1. Titanium wear particles activated this signaling pathway to the promoted expression of the ephrinB2 gene. However, ephrinB2 protein can be activated by osteoblast membrane receptor ephB4 to inhibit osteoclast differentiation. In in vivo experiments, we found that ephB4 could regulate Ti particle-mediated imbalance of OPG/RANKL, and the most important finding was that ephB4 relieved the release of proinflammatory factors. The ephB4-Fc inhibits wear particle-mediated osteolysis and inflammatory response through the ephrinB2/EphB4 bidirectional signaling pathway, and ephrinB2 ligand is expected to become a new clinical drug therapeutic target.

## 1. Introduction

Arthroplasty has been used clinically for decades and has become one of the preferred methods to treat serious joint diseases, benefitting millions of patients each year [1]. However, aseptic loosening is still a major cause of failure in total joint replacement [2]. It has been demonstrated that inflammation caused by wear particles such as titanium (Ti), ceramics, and polymethyl methacrylate (PMMA) and the subsequent periprosthetic osteolysis is the main pathological mechanisms leading to aseptic loosening [3]. The effects of wear particles on bone remodeling have been studied at the molecular level; for example, wear particles activate many osteoclast-related signaling pathways such as CN/NFAT, NF-*κ*B, ERK-MAPK, and AKT [2, 4–6]. The activity of wear particles can promote the differentiation of preosteoclasts into mature osteoclasts and enable their bone resorption function by activating the corresponding cell signaling pathways. This signaling pathway will be the focus of future research on aseptic loosening prevention and treatment [1, 3, 7].

The process of bone reconstruction is a dynamic balance of osteoblast-mediated bone formation and osteoclast-mediated bone resorption [8]. The communication between osteoblasts and osteoclasts plays a key role in bone remodeling [9, 10]. Over the past few decades, most researchers have focused on the development of a few types of cells, such as osteoclasts (ERK-MAPK signaling pathway [11], NF-*κ*B cell signaling pathway [12], and AKT cell signaling pathway [13]) or osteoblasts (WNT cell signaling pathway [14]); however, the communication between osteoclasts and osteoblasts has been largely neglected. In recent years, RANKL/OPG (receptor activator of nuclear factor *κ*B (NF-*κ*B) ligand/osteoclastogenesis inhibitory factor) of osteoblasts or bone cells has been found to be the main cytokine affecting the biological behavior of osteoclasts [15, 16]. The OPG-RANKL-RANK system plays an important role in bone metabolism. The main mechanism is the RANKL protein expressed by osteoblasts, which binds to the RANK protein expressed by osteoclast precursor or mature osteoclast. It promotes the differentiation and maturation of osteoclasts and enhances their activity. Osteoblasts also secrete OPG proteins, which compete with RANKL ligands for binding to RANK receptors, thus, blocking the signal transduction of RANKL ligands and RANK receptors, ultimately inhibiting osteoclast differentiation and maturation. The relative levels of RANKL and OPG expression in the bone microenvironment are the key to determining osteoclast formation and activity. If the expression level of the RANKL gene is higher than that of OPG, osteoclast formation is active. Conversely, osteoclast formation is inhibited. However, recent, growing evidence suggests that the direct contact interaction between osteoblasts and osteoclasts is also a key factor that influences the differentiation of osteoclasts and promotes the differentiation of osteoblasts. Furthermore, the communication is now thought to be bidirectional [9, 10, 17–19].

Eph receptors are membrane-bound proteins that form the largest family of tyrosine kinase receptors, are located on the cell membrane, and are divided into two classes [20–22]. Ephrin proteins are ligands of eph receptors and are divided into two types, namely, A and B [21]. Previous research related to eph/ephrin signaling mainly focused on neurological [23], hematological [24], and gastroenterological aspects. Research studies on the topic of osteoarthritis are scarce. This role of bidirectional ephrinB2/ephB4 signaling has recently been studied in the field of bone research. EphrinB2 ligand and ephB4 receptor are simultaneously expressed on the osteoblast membrane, and EphrinB2 ligand is also expressed on the osteoclast membrane [17, 22, 25, 26]. When the ligand binds to the receptor, both downstream signaling pathways are activated. The binding of ephB4 on the membrane of osteoblasts binds to ephrinB2 on the membrane of osteoclasts; it activates downstream signaling pathways and inhibits the maturation of osteoclasts, which is called “Reverse Signal”. Conversely, when ephrinB2 binds to ephB4 on the osteoblast membrane, it also promotes the mineralization of osteoblasts, which is known as “Forward Signal” [21].

Based on previous data, we speculate that ephrin-B2/ephB4 signaling may be an important pathway for bone remodeling imbalance around the prosthesis. The purpose of this study was to verify whether titanium particle-mediated osteolysis is inhibited by activated ephrin-B2 both in vitro and in vivo and to determine whether it represents a potential therapeutic target for the treatment of aseptic loosening.

## 2. Materials and Methods

### 2.1. Reagents

A penicillin-streptomycin solution, *α*-minimum essential medium (*α*-MEM), and fetal bovine serum (FBS) were obtained from Gibco; Thermo Fisher Scientific, Inc., (Waltham, MA, USA). Soluble recombinant mouse macrophage-colony stimulating factor (M-CSF), recombinant mouse RANKL, recombinant mouse ephrinB2-Fc, recombinant mouse ephB4-Fc (erythropoietin-producing human hepatocellular receptor 4), Fc fragments, and anti-Ig-Fc were obtained from R&D Systems, Inc., (Minneapolis, MN, USA). Tartrate-resistant acid phosphatase (TRAP) and alkaline phosphatase (ALP) were purchased from Sigma-Aldrich and Merck KGaA (Darmstadt, Germany). High purity Ti particles (average diameter. 3-5 *μ*M) were obtained from Johnson Matthey (cat. no., 00681; London, UK). Dexamethasone, ascorbic acid, and *β*-glycerol phosphate sodium were obtained from Sigma-Aldrich and Merck KGaA (Darmstadt, Germany). The common antibodies, GAPDH, C-FOS, NFATc1, TRAP, and cathepsin K (CK), were purchased from Cell Signaling Technology, Inc., (Danvers, MA, USA). Ephrin-B2 antibodies were purchased from R&D Systems. Enzyme-linked immunosorbent assay (ELISA) kits for detecting mouse interleukin- (IL-) 6, IL-1*β*, tumor necrosis factor (TNF)-*α*, and IL-10 were purchased from R&D Systems, Inc.

### 2.2. Preparation of Ti Particles

Ti particles were prepared as described previously [1]. We followed the methods of Dr. Ge et al. 2018 [27]. The wear particles were soaked in 75% (*v*/*v*) ethanol for 48 hours to remove endotoxins [27]. A chromogenic end-point TAL with Diazo coupling kit (Xiamen Houshiji, Fujian, China) was used to detect endotoxins [27]. The wear particles can be used for experiments until the endotoxin unit (EU) concentration is <0.1 EU/mL [27]. This concentration was similar to that of the wear particles around the prosthesis, as reported previously [1, 7].

### 2.3. Cell Culture

20 healthy female C57BL/6 mice (16 g-18 g/per) were obtained from the Animal Center Research Committee of the Shanghai Ninth People's Hospital (Shanghai, China). All animal procedures were approved by the Animal's Hospital affiliated to Shanghai Jiao Tong University. Bone marrow macrophages (BMMs) were collected from the tibias and femurs of 4-6-week-old C57BL/6 mice, containing 30 ng/mL M-CSF, 10% FBS, and 1% penicillin-streptomycin at 37°C for 3 days [27]. At ~80% confluence, the cells were dissociated with 0.25% (*v*/*v*) trypsin and then plated onto culture plates. For cocultures, BMMs were seeded at a density of 5 × 10^5^ cells in 12-well plates containing 5 × 10^4^ MC-3T3-E1 cells in 1 mL *α*-MEM supplemented with 10% FBS, 10^−8^ M dexamethasone, 50 mg/L ascorbic acid, and 10 mM *β*-glycerol phosphate sodium [27]. In order to stimulate reverse signaling, soluble recombinant mouse ephB4-Fc and Fc fragments (clustered with anti-Fc antibody) were added to the medium at a concentration of 4 *μ*g/mL [27].

### 2.4. Alkaline Phosphatase (ALP) Staining and Alizarin Red Staining

To detect the level of osteogenic function, MC3T3-E1 cells (1 × 10^5^ cells/well) were seeded on 24-well plates in triplicate, containing 10^−8^ M dexamethasone, 50 mg/L ascorbic acid, and 10 mM *β*-glycerol phosphate sodium [27]. The MC3T3-E1 cells were fixed with 4% PFA. After 7 days, The MC3T3-E1 cells were stained using an ALP kit (Renbao, Shanghai, China) and observed by optical microscopy (magnification, ×10). After 21 days of culture, Alizarin Red S staining (Sigma-Aldrich; Merck KGaA) was used to assessed calcium deposition.

### 2.5. TRAP Staining

BMMs (1 × 10^4^ cells/well) were seeded onto 96-well plates in triplicate, containing 30 ng/mL M-CSF and 50 ng/mL RANKL. After 7 days' culture, 4% PFA was used to fix osteoclasts for 20 min at 37°C. The osteoclast activity was detected with the TRAP staining kit (Sigma-Aldrich; Merck KGaA) according to the manufacturer's protocol. If the number of nuclei was >3, it was considered TRAP positive. Image-Pro Plus 6.0 (Media Cybernetics, Inc., Rockville, MD, USA) was used to quantify the total area of mature osteoclasts.

### 2.6. F-Actin Ring Formation Assay and Bone Pits Resorption Assay

BMMs (1 × 10^4^ cells/well) were seeded onto 96-well plates in triplicate, containing 30 ng/mL M-CSF and 50 ng/mL RANKL. After 7 days' culture, 4% PFA was used to fix osteoclasts for 20 min at 37°C [27]. The rhodamine-conjugated phalloidin (Cytoskeleton, Inc., Denver, CO, USA) was used to detect the cytoskeleton (F-actin ring). BMMs were stained at 37°C for 1 h, and then, washed with PBS three times, each time for 10 min. A LSM5 confocal microscope (magnification, ×10; Carl Zeiss AG, Oberkochen, Germany) was used to observe the F-actin ring. The images were analyzed using the Image-Pro Plus 6.0 software. Sterile bone pieces from the 4 groups were placed into 96-well plates, containing BMMs (1 × 10^4^ cells) with 30 ng/mL M-CSF and 50 ng/mL RANKL. After 12 days, the 0.25% (*v*/*v*) trypsin was used to digest the cells on the bone pieces, and the cells were then washed 3 times with PBS. A Quanta 250 scanning electron microscope (SEM; FEI; Thermo Fisher Scientific, Inc.) with a magnification of 10 kV was used to obtain on the bone surface. The resorption area was measured using Image-Pro Plus 6.0.

### 2.7. Small Interfering C-Fos RNA and Small Interfering NFATc1 RNA

The siRNA specific for C-fos, NFATc1, and scrambled siRNA were purchased from RiboBio Company (RiboBio, Guangzhou, China). The RNAiMAX (Thermo Fisher Scientific, Woburn, MA, USA) and SiRNA were used to transfect into BMMs according to the manufacturer's protocol.

### 2.8. Immunofluorescence Staining of Ephrin-B2 Localization

The sample was processed as described previously. Briefly, the sample was divided into 6 groups: Si-C-fos con, Si-C-fos RNA1, Si-C-fos RNA2, Si-NFATc1 con, Si-NFATc1 RNA1, and Si-NFATc1 RNA2. After 7 days, they were fixed with 4% paraformaldehyde for 20 min at 37°C, washed 2-3 times with PBS for 10 min, and incubated (37°C) with 5% bovine serum albumin (cat. no. A602440; Sangon Biotech Co., Ltd., Shanghai, China) for 60 min. Finally, a mouse anti-ephrin-b2 monoclonal antibody (cat. no. ab150411; 1: 250; Abcam, Cambridge, UK) was added to the plates at 4°C for 12 h. Goat Anti-Rabbit IgG (Alexa Fluor® 488; cat. no. 111-547-008; Jackson ImmunoResearch Laboratories, Inc., West Grove, PA, USA) conjugated with Fluor® 488 (1 : 250) at room temperature for 1 h in darkness. An LSM5 confocal microscope (magnification, ×40) was used to observe the localization of ephrinB2.

### 2.9. Enzyme-Linked Immunosorbent Assay (ELISA)

BMMs (1 × 105 cells/well) were seeded onto 6-well plates, containing 30 ng/mL M-CSF and 50 ng/mL RANKL. Four cell groups: including BMMs; BMMs+Ti (0.1 mg/mL); BMMs+ephB4-Fc (4 *μ*g/mL); and BMMs+ephB4-Fc (4 *μ*g/mL) +Ti (0.1 mg/mL). After 24 h culture, ELISA kits (R&D Systems, Inc., Minneapolis, MN, USA) were used to assess the concentrations of interleukin- (IL-) 6, IL-1*β*, tumor necrosis factor- (TNF-) *α*, and IL-10 according to the manufacturer's guidelines.

### 2.10. EphrinB2 Knockdown and Overexpression

The following lentiviral vectors containing small hairpin RNA (shRNA) against ephrinB2 were used: (sh1-ephrinB2: 5′-*GCAGACAGATGCACAATTA*-3′; sh2-ephrinB2: 5′-*CCAGACCAAGATGTGAAAT*-3′; sh3-ephrinB2:5′-*GCTAGAAGCTGGTACAAAT-*3′; NC-ephrinB2:5′-TTCTCCGAACGTGTCACGT-3′).

The coding sequences of m-ephrinB2 (over-ephrinB2: Gene ID: NM_010111) and scrambled control (Tongke, Shanghai, China) were used to transfect Raw264.7 cells. Vectors were propagated in HEK293 cells, purified, and titration of the particles was performed by the optical absorbance. Next, 5 *μ*g/mL of puromycin (Sigma-Aldrich) was added to RAW264.7 cells at 48 h postinfection and maintained in 2 *μ*g/mL puromycin to remove Raw264.7 cells that lost their shRNA and overexpression.

### 2.11. Reverse Transcription Quantitative Polymerase Chain Reaction (RT-qPCR)

The sample was processed as described previously. Briefly, total RNA was collected using an RNeasy Mini kit (Qiagen, Inc., Valencia, CA, USA) and reverse transcribed into cDNA (Takara Bio, Inc., Otsu, Japan). The SYBR Premix Ex Taq kit (Takara Biotechnology Co., Ltd.) and an ABI 7500 Sequencing Detection System (Applied Biosystems; Thermo Fisher Scientific, Inc.) was used to perform qPCR. The following thermocycling conditions were used: 40 cycles of denaturation at 95°C for 5 s and amplification at 60°C for 24 s. GAPDH was used as the reference gene, and all the reactions were run in triplicate. The PCR primers were designed as follows:

GAPDH F, 5′-CACCACCATGGAGAAGGCCG-3′; GAPDH R, 5′-ATGATGTTCTGGGCAGCCCC-3′; OPG F,5′-CGAGCGCAGATGGATCCTAA-3′; OPG R, 5′-CCACATCCAACCATGAGCCT-3′; RANKL F, 5′-CCCATCGGGTTCCCATAAAGT-3′; RANKL R, 5′-CGACCAGTTTTTCGTGCTCC-3′; RUNX2 F, 5′-TCGGAGAGGTACCAGATGGG-3′; RUNX2 F, 5′-TGAAACTCTTGCCTCGTCCG-3′; Collagen 1 F, 5′- GAGAGGTGAACAAGGTCCCG-3′; Collagen 1 R, 5′- AAACCTCTCTCGCCTCTTGC-3′; EphrinB2 F, 5′-AAT CTCCTG GGT TCCGAAGT-3′; EphrinB2 R, 5′-GTCTCCTGCGGT ACT TGA GC-3′.

### 2.12. Western Blot Analysis

Cells were seeded at a density of 4 × 10^6^ cells into 6-well plates with Ti particles (30 ng/mL M-CSF, 50 ng/mL RANKL), including the following groups of comparison: Si-C-fos con, Si-C-fos RNA1, Si-C-fos RNA2; Si-NFATc1 con, Si-NFATc1 RNA1, Si- NFATc1 RNA2 for 48 h and OE-ephrinB2-control, OE-ephrinB2; sh-ephrinB2-control, and sh-ephrinB2-Mix for 5 days. Then, radioimmunoprecipitation assay (RIPA) lysis buffer (cat. no. C500005; Sangon Biotech Co., Ltd.) containing 1 *μ*M protease inhibitor was added to cells for 15 min and centrifuged (4°C) at 12,000 ×g for 10 min, and the supernatant was collected. The bicinchoninic acid assay (BCA) was measured the total protein concentration. Equal amounts of the protein lysates were separated via SDS-PAGE (10% gel), and gels were transferred to polyvinylidene difluoride membranes, blocked for 1 h with 5% (*w*/*v*) milk, and incubated overnight at 37°C with primary antibodies against GAPDH (cat. no. #8884; 1 : 1,000; Cell Signaling Technology, Inc.), c-Fos (cat. no. #2250; 1 : 1,000; Cell Signaling Technology, Inc.), NFATc1 (cat. no. #8032; 1 : 1,000; Cell Signaling Technology, Inc.), and TRAP (cat. no. ab133238; 1 : 1,000; Abcam, Cambridge, UK) overnight. The horseradish peroxidase-conjugated secondary antibodies (cat. no. #7074; 1: 5,000; Cell Signaling Technology, Inc.) reactivity was detected by the Odyssey infrared imaging system (LI-COR Biosciences, Lincoln, NE, USA).

### 2.13. Animals and Animal Procedures

A mouse calvarial osteolysis model was built to measure the antiosteolytic suppressing effect of EphB4-Fc in vivo. All animal procedures were approved by the Animal Hospital affiliated to Shanghai Jiao Tong University. Twenty healthy female C57BL/6 mice (16 g-18 g, each) were assigned randomly to 4 groups: PBS control (sham), Ti (vehicle, 30 mg), Ti (30 mg) with low (3 *μ*g/2d) concentrations of ephB4-Fc, and Ti (30 mg) with high (7 *μ*g/2d) concentrations of ephB4-Fc for 14 day. After the end of the experiment, the mice were euthanized. The skull was removed and soaked in 4% paraformaldehyde for micro-CT (Skyscan 1072; Skyscan, Aartselaar, Belgium) analysis. An isometric resolution of 9 mm was set and X-ray energy settings were 80 kV and 80 mA. Bone volume/tissue volume (BV/TV) and bone mineral density (BMD) were measured as reported previously.

### 2.14. Immunohistochemistry and Immunofluorescence

After that, samples were decalcified in decalcification solution (10% ethylenediaminetetraacetic acid EDTA) for 7 days and then embedded in paraffin. Histological sections were prepared for H&E staining, TRAP staining, and immunohistochemistry of ephrinB2, IL-1*β*, TNF-*α*, IL-6, and immunofluorescence of OPG, RANKL, and then observed in a light microscopy (magnification, ×10). Images were analyzed using Image-Pro Plus 6.0 (Media Cybernetics, Inc., Rockville, MD, USA). In addition, periprosthetic interface tissues were gained at the time of revision surgery. This study was approved by the local Ethics Committee. The sample was embedded in paraffin. Histological sections were prepared for immunohistochemistry. Antibody ephrinB2 (human, 1 : 200), Antibody IL-1*β* (human, 1 : 200), antibody TNF-*α* (human, 1 : 200), and antibody IL-6 (human, 1 : 200) were used for immunohistochemistry.

### 2.15. Statistical Analysis

Data are expressed as mean ± standard deviation. Differences among groups were analyzed by one-way analysis of variance and the post hoc tests with the Student-Newman-Keuls post hoc test using the SPSS software (version 11.0; SPSS Inc., Chicago, IL, USA). *P* < 0.05 was considered to indicate a statistically significant difference.

## 3. Results

### 3.1. Establishment of a Coculture Model and Effect on Osteogenic Differentiation

**Figure 1:**
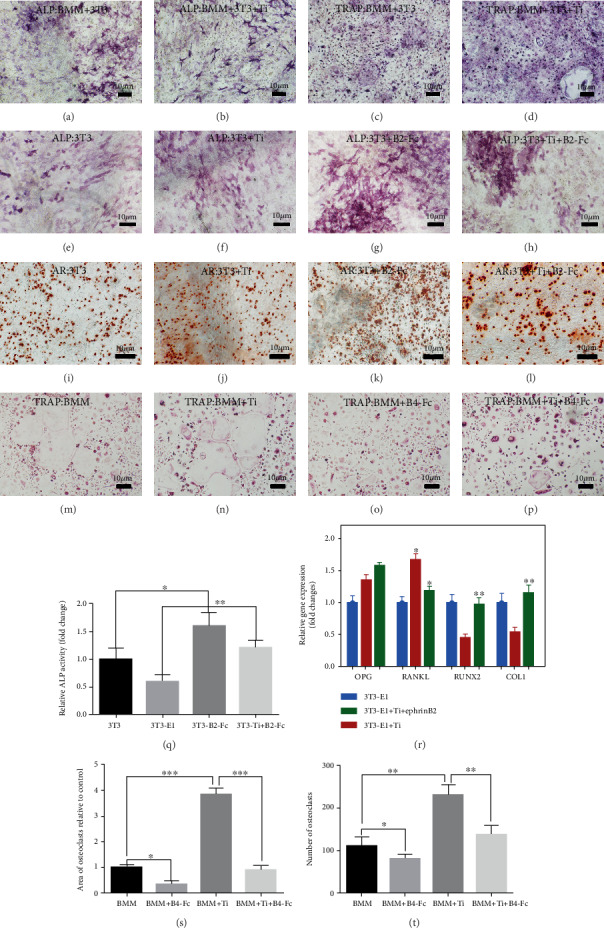
The effect of coculture of osteoblasts and osteoclasts on cell differentiation with or without Ti. ALP staining and TRAP staining under conditions of direct coculture of BMMs and 3T3-E1 cells with or without Ti (a–d). (e–l) Show ALP staining and Alizarin Red staining (AR) for simulated coculture with or without Ti. (m–p) Show TRAP staining for simulated coculture with or without Ti. (q) Shows a quantitative analysis of ALP staining. (r) Shows the effect on osteoblast differentiation under coculture conditions. (s, t) Show a quantitative analysis of the number and area of osteoclasts. Data are represented as mean ± standard deviation, *n* = 3 (^∗^*P* < 0.05, ^∗∗^*P* < 0.01).

Figures 1(a) and 1(b) indicated ALP staining under direct coculture conditions with or without Ti. Figures 1(c) and 1(d) indicated TRAP staining (BMMs) under direct coculture conditions with or without Ti. Direct coculture revealed that titanium particle-mediated osteoclast differentiation could be inhibited. Figures 1(e)–1(l) show alkaline phosphatase (ALP, 3T3-E1) staining and Alizarin Red (AR, 3T3-E1) staining under ephrinB2-Fc conditions with or without Ti. This part of the experiment showed that the wear particles inhibited the differentiation of osteoblasts. But after the addition of ephrinB2-Fc, it alleviated the differentiation and maturation of osteoblasts.  Figure 1(q) shows a quantitative analysis of the alkaline phosphatase activity of osteoblasts. We found that after the addition of ephrinB2-Fc, osteoblast differentiation was promoted and osteogenic related genes (*RANKL*, *RUNX2*, and *COL1*) were also upregulated by PCR (Figure 1(r)). Figures 1(m)–1(p) show TRAP staining upon ephB4-Fc treatment with or without Ti particles. Figures 1(s) and 1(t) show the quantitative analysis of the number of osteoclasts and the area of osteoclasts. We came to a conclusion similar to that reached through direct cocultivation experiments: the differentiation of osteoclasts was significantly inhibited upon addition of ephB4-Fc.

### 3.2. Effects on Bone Resorption and F-Actin of Osteoclasts

**Figure 2:**
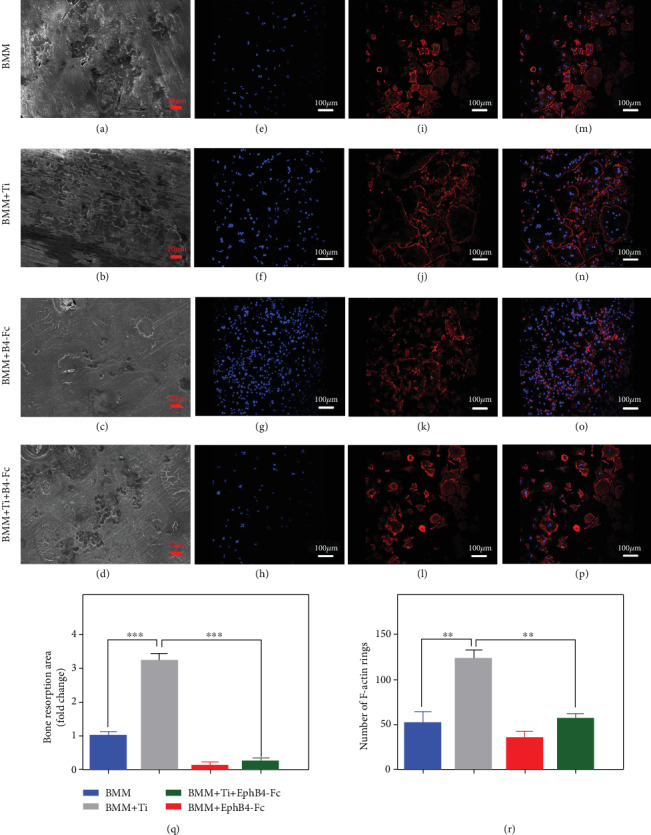
The effect of coculture of osteoblasts and osteoclasts on osteoclast function with or without Ti. (a–d) Show bone absorption experiment with or without ephB4-Fc or Ti in the presence of M-CSF and RANKL for 12 days. (e–o) Show the F-actin rings with or without ephB4-Fc or Ti. (p) Shows the histogram of the total area of bone resorption was based on the size of the above pits. (q) Shows the number of F-actin rings in the 5 randomized fields. Data are represented as mean ± standard deviation, *n* = 3 (^∗^*P* < 0.05, ^∗∗^*P* < 0.01).

In order to further verify the function of osteoclasts in the presence of Ti particles, we performed bone pit absorption experiments (Figures 2(a)–2(d)) by scanning electron microscopy and F-actin experiments (Figures 2(e)–2(o)) by observation in laser scanning confocal microscope. The following groups were compared: BMMs; BMMs+Ti (0.1 mg/mL); BMMs+ephB4-Fc (4 *μ*g/mL); and BMMs+ephB4-Fc (4 *μ*g/mL) +Ti (0.1 mg/mL). We found that Ti particle-induced bone absorption is significantly inhibited upon addition of ephB4-Fc (Figure 2(p)), and Ti particle-induced formation of F-actin was also significantly inhibited (Figure 2(q)). Experiments have shown that ephB4-Fc can inhibit the Ti particle-induced differentiation of preosteocytes to mature osteoclasts.

### 3.3. The EphrinB2 Gene Is Located Downstream from the c-Fos/NFATc1 Gene, and EphrinB2 Is Located on the Surface of the BMM Membrane

**Figure 3:**
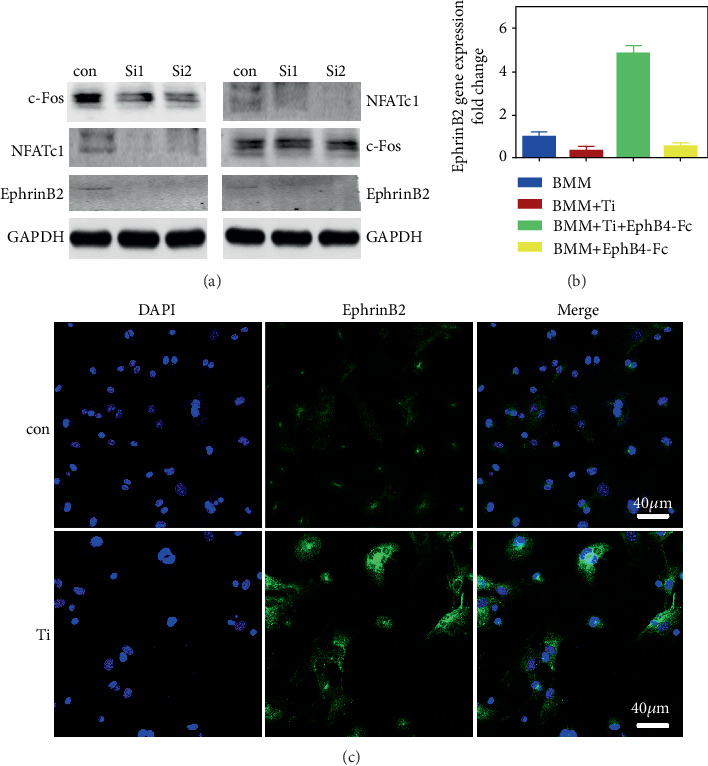
The expression of ephrinB2 is mediated by c-Fos/NFATc1 signaling pathway. (a) Shows that the ephrinB2 gene is regulated by the c-Fos/NFATc1 signaling pathway through sic-Fos RNA and siNFATc1 RNA. We find that it promotes high expression of ephrinB2 gene and protein with Ti (b, c) and the ephrinB2 protein is located on the surface of the BMMs membrane by immunofluorescence. Data are represented as mean ± standard deviation, *n* = 3 (^∗^*P* < 0.05, ^∗∗^*P* < 0.01).

Next, we verified the relationship between ephrinB2, c-Fos, and NFATc1 by small interfering c-Fos RNA and small interfering NFATc1 RNA in the present of Ti particles (“Si1” and “Si2” are two different small interfering RNAs we selected for c-Fos and NFATc1).  Figure 3(a) shows that the expression of NFATc1 and ephrinB2 proteins was inhibited after the addition of small interfering c-Fos RNA. The ephrinB2 protein was also inhibited after the addition of small interfering NFATc1 RNA. However, c-Fos protein levels were not changed. Finally, we found by immunofluorescence that ephrinB2 protein was distributed on the cell surface (Figure 3(c)). The most important finding was that the ephrinB2 protein expression was significantly increased after the addition of Ti particles compared with the control group (Figure 3(b)).  Figure 3 illustrates that the Ti particle can activate the C-fos/NFATc1 signaling pathway to further activate the ephrinB2 gene, and that the Ti particle-induced ephrinB2 protein regulated by c-Fos/NFATc1 was transported to the surface of the cell membrane to continue to function as a membrane ligand.

### 3.4. Effects of EphB4-Fc on Osteoclastogenesis-Related Protein

**Figure 4:**
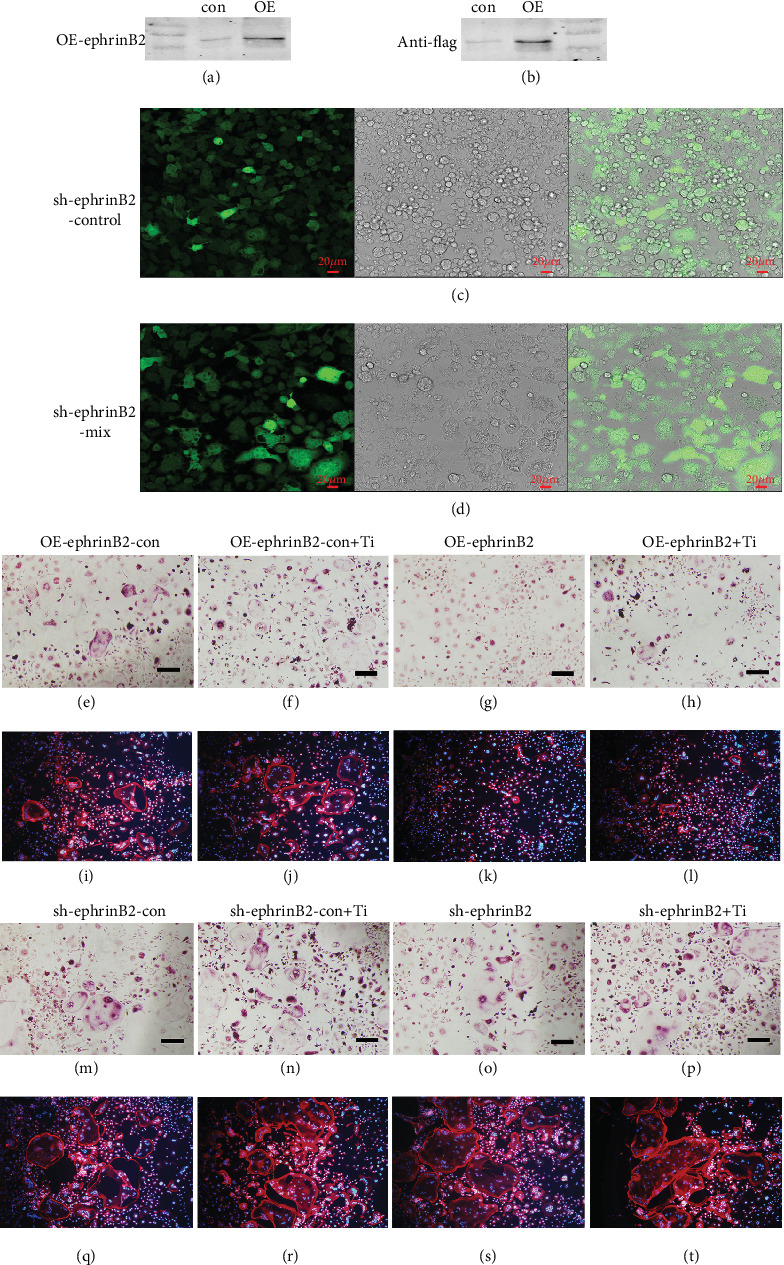
The function of osteoclast differentiation was verified by overexpressing and silencing the ephrinB2 gene. (a, b) Show that the high expression of ephrinB2 gene is verified on the raw264.7 cell membrane surface by lentiviral transfection technology by anti-ephrinB2 and anti-flag. (c, d) Show that the low expression of ephrinB2 gene is verified on the raw264.7 cell membrane surface by GFP. (e–t) Show osteoclast differentiation and function are significantly inhibited by TRAP and rhodamine staining with Ti.

We found that ephrinB2 was significantly upregulated after the addition of Ti particles. In past years, scholars have discovered that Ti particles activate the c-Fos/NFATc1 signaling pathway, and Figure 3 shows that the *ephrinB2* gene was also regulated by the c-Fos/NFATC1 signaling pathway. By overexpressing and knocking down the ephrinB2 gene, we investigated whether the formation of osteoclasts was inhibited in the presence of Ti particles. First, we first established a cell line (Raw264.7) that overexpresses ephrinB2 (Figures 4(a) and 4(b)) and knocks down ephrinB2 (Figures 4(c) and 4(d)). We found that ephrinB2 and flag proteins were expressed in cells overexpressing ephrinB2 by Western blotting, but not in the control group (Figures 4(a) and 4(b)). When we designed the knocking down ephrinB2 cell line, we inserted GFP green fluorescence in into plasmid. So the green fluorescence intensity represents the efficiency of transfection. By observing the green fluorescent cells and the cells under the ordinary microscope, we found that the cells in the knocking down group and the control group could express green fluorescence under the excitation light (Figures 4(c) and 4(d)). All of the above experiments demonstrate that we have established reliable overexpression of ephrinB2 and knockout of ephrinB2 cell lines. Next, as shown in Figures 4(e) and 4(f), the formation of osteoclasts and F-actin was significantly inhibited with or without Ti particles in the overexpression group following the addition of ephB4-Fc. In contrast, in the knockdown group, we found that osteoclasts were not inhibited (Figures 4(g) and 4(h)). In the overexpressed ephrinB2 group, proteins (NFATc1, Cathepsin k, MMP9, and TRAP) were all inhibited. However, compared with the control group, the expression of osteoclast-related protein was increased in the knockout group (Supplementary Figure S3).

### 3.5. Effects of EphB4-Fc on Proinflammatory Factors

**Figure 5:**
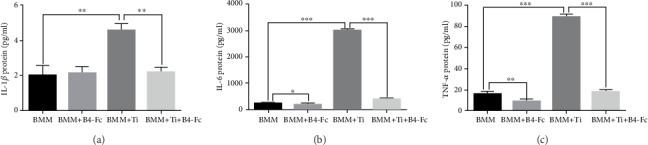
The effect of inflammatory factors on coculture environments with or without Ti. ELISA conducted to evaluate (a) IL-1*β*, (b) IL-6, and (c) TNF-*α*. Data are presented as mean ± standard deviation. *n* = 3^∗^*P* < 0.05 and ^∗∗^*P* < 0.01.

Several researchers have encountered an important problem: upon addition of Ti particles, macrophages secrete proinflammatory factors, which promote the differentiation of macrophages into osteoclasts [11, 15, 28, 29]. We further tested the supernatant of macrophages through ELISA. We compared four groups: BMMs, BMMs+B4-Fc, BMMs+Ti, and BMMs+Ti+B4-Fc. Figures 5(a)–5(c) show that IL-1*β*, TNF-*α*, and IL-6 increased significantly upon the addition of Ti particles. However, IL-1*β*, TNF-*α*, and IL-6 were significantly decreased upon adding ephB4-Fc. The above experiments demonstrate that ephB4-Fc can inhibit the release of proinflammatory factors mediated by Ti particles. Thereby, the formation of osteoclasts mediated by inflammatory factors is indirectly inhibited.

### 3.6. Effects of EphB4-Fc on Ti Particle-Induced Osteolysis In Vivo

**Figure 6:**
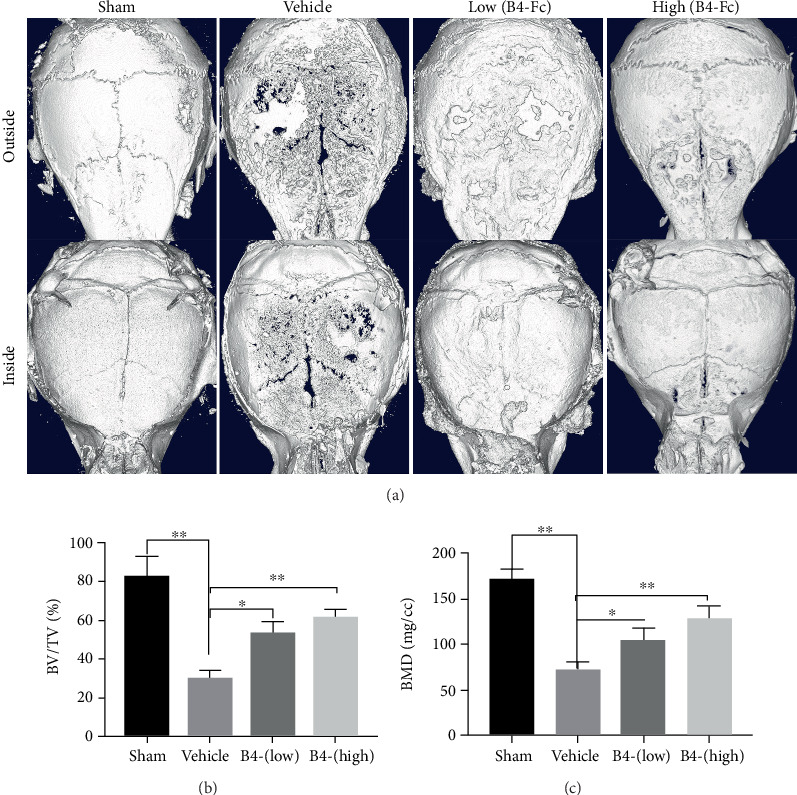
Histomorphometric analysis of calvaria in mice treated with low and high ephB4-Fc. (a) Representative microcomputerized tomography (*μ*CT) 3D reconstructed images of calvaria for each group, (b) bone volume to tissue volume (BV/TV), (c) bone mineral density (BMD). *n* = 3^∗^*P* < 0.05 and ^∗∗^*P* < 0.01.

Previous in vitro studies have shown that ephB4-Fc can inhibit the formation of osteoclasts and the secretion of proinflammatory factors in the early stage via bone marrow macrophages. Therefore, in order to further verify this osteolysis mediated by Ti particles in vivo, we established a Ti particle-induced osteolysis model. Extensive bone resorption was observed in the Ti by microcomputed tomography (CT) with three-dimensional reconstruction. Ti particle-mediated osteolysis was significantly inhibited by ephB4-Fc treatment in a dose-dependent manner (Figure 6(a)). Bone volume/total volume (BV/TV) and bone mineral density (BMD) were measured from three-dimensional reconstruction images as previously reported (Figures 6(b) and 6(c)). When ephB4-Fc was given at 2 *μ*g/per and 4*μ*g/per daily, osteolysis was inhibited compared with the vehicle group.

**Figure 7:**
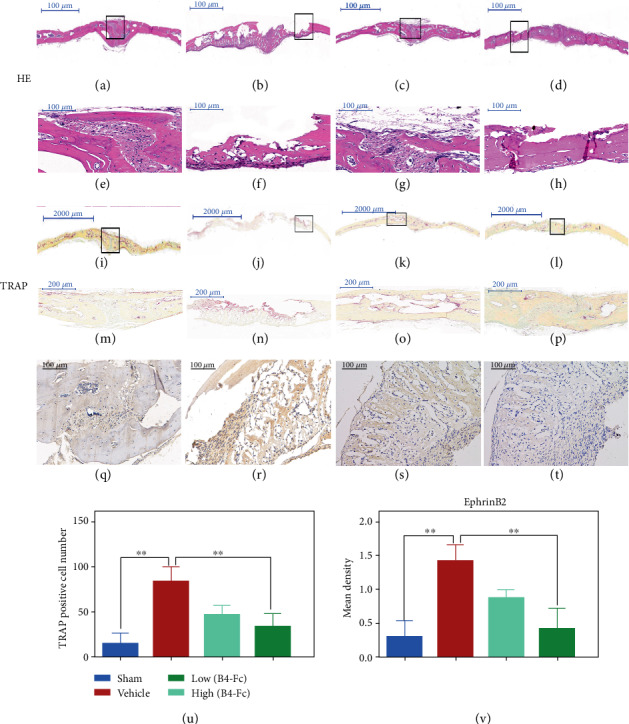
Histological staining of calvaria sections and immunohistochemistry of ephrinB2. (a–p) Show that hematoxylin and eosin (H&E) and tartrate-resistant acid phosphatase- (TRAP-) stained with low and high ephB4-Fc. (q–t) Show that the expression of ephrinB2 protein with low and high ephB4-Fc. (u) Quantitative analysis of the number of TRAP-positive multinuclear macrophages on the surface of the sections. (v) Quantification of positive staining and mean density of positive staining. Data are presented as mean ± standard deviation. *n* = 3^∗^*P* < 0.05 and ^∗∗^*P* < 0.01.

To further confirm that ephB4-Fc could reduce Ti particle-mediated osteolysis in vivo, we performed histological assessment and histomorphometric analysis. The H&E staining showed a large amount of accumulation of proinflammatory macrophages in the vehicle group, and marked osteolysis was also found to occur around the wear particles. Next, we again further performed TRAP staining. The results showed that TRAP-positive macrophages aggregated around the wear particles and released H+ further damaging the bone matrix. However, upon treatment with ephB4-Fc, Ti particle-mediated osteolysis was significantly inhibited (Figures 7(a)–7(p)).

In order to further validate the effect of wear particles on ephrinB2, we performed an immunohistochemistry experiment. The expression of the ephrinB2 protein was significantly higher in the vehicle group expressed than in the sham group. However, upon treatment with ephB4-Fc, we found that the expression of ephrinB2 protein was significantly lower than in the vehicle group (Figures 7(q)–7(t)).

### 3.7. Effects of EphB4-Fc on OPG/RANKL and Reduced Expression of TNF-*α*, IL-1*β*, and IL-6

**Figure 8:**
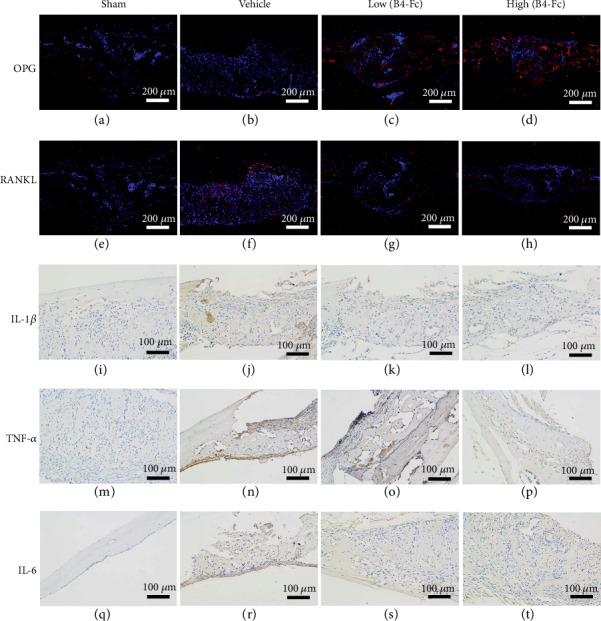
The effect on the balance of OPG/RANKL and the release of inflammatory factors in vivo. (a–h) Show that the expression of the OPG/RANKL protein was measured by immunofluorescence on tissue sections in the presence of low and high ephB4-Fc. (i–t) The expression of the IL-1*β*, TNF-*α*, IL-6 protein by immunohistochemistry.

The above experiments demonstrate that ephB4-Fc inhibits the differentiation of osteoclasts and the bone resorption function through the ephrinB2 signaling pathway. Thus, we investigated whether ephB4-Fc has an effect on the expression of OPG/RANKL in osteoblasts. We further performed an immunofluorescence analysis of OPG/RANKL. Compared with the sham group, the expression of OPG was significantly reduced and the expression of RANKL was significantly increased in the vehicle group. However, compared with the vehicle group, the expression of OPG protein increased and the expression of RANKL protein decreased after the addition of ephB4-Fc (Figures 8(a)–8(h)). The results indicate that eph has an effect on the expression of osteogenic OPG/RANKL, and that the OPG/RANKL ratio is important for osteoclast differentiation. We verified that ephB4-Fc inhibited the release of proinflammatory factors in vitro, and we conducted the corresponding studies in vivo. We investigated the expression of IL-1*β*, TNF-*α*, and IL-6 proteins by immunohistochemistry (Figures 8(i)–8(t)) and found that the three of them were highly expressed in the vehicle group. However, upon the addition of ephB4-Fc, the release of inflammatory factors was significantly inhibited, and the effect of inhibition was significantly dose-dependent.

## 4. Discussion

Aseptic loosening caused by osteolysis around the prosthesis remains one of the most common complications of joint replacement (Supplementary Figure S1) [28, 30–32]. So far, researchers have not been able to prevent this complication through the structural design of the implant or through surface modification of the implant [33, 34]. However, these methods are less likely to eliminate particle generation on the bearing surface. The cause of the wear particles is attributed to the long-term wear of the bearing surface of the load-bearing part, which exacerbates the aggregation of macrophages and the release of inflammatory factors [35]. Furthermore, while new materials may help in the prevention of osteolysis, they are of no help against already existing osteolysis. Although the precise underlying mechanisms are still unclear, the chronic inflammation induced by wear particles and the imbalance of bone remodeling between osteoblasts and osteoclasts caused by wear particles remain an important issue for the cause of aseptic loosening [3].

Previous studies have indicated that the eph/ephrin signaling pathway regulates bone regeneration by direct contact [9, 17]. The unit composed of osteoblasts and osteoclasts is an essential part in regulating bone remodeling [22].The existence of such a unit in vivo cannot be refuted. The eph receptor family, the most common subfamily of the tyrosine protein kinase receptor, and its ligands are proteins of the ephrin family. The binding of the ephB4 receptor on the osteoblast membrane to the ephrinB2 ligand of the osteoclast membrane inhibits the formation of osteoclasts and promotes the differentiation of osteoblasts in vitro. However, the role of bidirectional signaling in Ti particles-mediated osteolysis remains unclear. We revealed by immunohistochemistry that inflammatory factors and ephrinB2 were significantly elevated around the prosthesis (Supplementary Figure S2.). The aim of this study was to understand wear particle-mediated osteolysis from the perspective of the bone multicellular (osteoblast/osteoclast) unit.

Previous studies have shown that wear particles can promote the accumulation of proinflammatory macrophages [28, 30]. Most importantly, such accumulation accelerates the differentiation of BMMs into osteoclasts. In the current study, it was found that Ti particles significantly increased the number of TRAP-positive cells (Figures 1(s) and 1(t)) and the expression of ephrinB2 gene (Figure 3(b)) and protein (Figure 7(v)). This result indicates that Ti promoted the differentiation of BMMs into osteoclasts and activated the ephrinB2 gene through the corresponding signaling pathway. We further validated this finding through interference with osteoclast-specific nuclear transcription factors with si-c-Fos RNA and si-NFATc1 RNA. We examined the expression of c-Fos protein, NFATc1 protein, and ephrinB2 protein by Western blot to verify their positional relationship. The signal path of c-Fos/NFATc1/ephrinB2 was thus determined. Previous studies have shown that Ti particles activate the c-Fos/NFATc1 signaling pathway. High expression of the ephrinB2 gene is certain to occur in the presence of Ti particles. As mentioned above, ephB4 receptors on the osteoblast membranes bind to ephrinB2 ligands on the osteoclast membrane, activates ephrinB2 ligands, and exert negative feedback control on c-Fos transcription, thereby, inhibiting the maturation of osteoclasts. However, Ti particles promote the high expression of ephrinB2 by the c-Fos/NFATc1 signaling pathway. The question remains as to whether the Ti particle-mediated osteoclastogenesis can be inhibited through bidirectional signaling?

Further, we overexpressed and knocked down the expression of ephrinB2 protein. And then, we observed the number of osteoclasts by TRAP staining. Rhodamine staining was used to observe F-actin formation and bone resorption. As shown in Figure 4, after the addition of ephB4-Fc, the differentiation and function of osteoclasts were significantly inhibited in the presence of Ti particles in the overexpression group. Moreover, in the Sh-ephrinB2-Mix+Ti group, we found that the differentiation of osteoclasts was not inhibited compared with the other groups, but the number of TRAP-positive cells was higher than in other groups. The experiments demonstrate that activated ephrinB2 can further inhibit Ti particle-mediated osteoclastogenesis via negative feedback inhibition of the c-Fos/NFATc1 signaling pathway.

In vivo, we demonstrated that Ti particle-mediated osteolysis can be significantly inhibited after the addition of a low-dose of ephB4-Fc and a high-dose of ephB4-Fc via micro-CT (Figure 6). We simulated the loosening of the prosthesis, and we also tested the effect of OPG/RANKL on osteoblasts. A large number of studies have shown that in the direct coculture system of osteoblasts and osteoclasts, the OPG/RANKL ratio is an extremely important factor for the regulation of osteoclast differentiation. The study found that the OPG/RANKL ratio was significantly upregulated after the addition of ephrinB2-Fc by immunofluorescence (Figures 8(a)–8(h)). In vitro, we also examined the expression of osteoblast-associated genes after adding ephrinB2-Fc by PCR (Figure 1(r)). We concluded that the OPG gene did not change significantly, whereas the expression of the RANKL gene was significantly reduced after the addition of ephrinB2-Fc. The OPG/RANKL ratio was significantly upregulated. This indicates that the ephrinB2 ligand of the osteoclast membrane downregulates the expression of osteoblast-associated RANKL, which can competitively bind to the RANK receptor (blocking the binding of OPG to the RANK receptor).

Previous studies have shown that Ti wear particles promote the release of inflammatory factors and thus affect the differentiation of osteoclasts. We then tested the proinflammatory factors using ELISA (Figure 5) and immunohistochemistry (Figures 8(i)–8(t)). We further found that the Ti-mediated release of proinflammatory factors was inhibited by the addition of ephB4-Fc.

## 5. Conclusion

The study found that the expression of ephrinB2 protein around aseptic loosening and inflammatory cytokines appear around the prosthesis. We verified the ephrinB2 protein produced through the c-Fos/Nfatc1 signaling pathway by small interfering c-Fos-RNA and small interfering Nfatc1-RNA. We activated ephrinB2 ligand around the prosthesis by adding ephB4-Fc and found that it could inhibit osteolysis (Supplementary Figure S4). We further validated this phenomenon by overexpressing and knockdown ephrinB2. Most importantly, we found the imbalance between OPG/RANKL mediated by wear particles can be significantly changed.

## Data Availability

The data used to support the findings of this study are available from the corresponding author upon request.

## Supplementary Materials

Supplementary materials Supplementary Figure S1: preoperative X-ray and postoperative prosthesis pictures. Supplementary Figure S2: immunohistochemistry of the interface membrane. Supplementary Figure S3: functionalization of osteoclast differentiation after overexpression and silencing of Raw264.7 cells was verified by western blot. Supplementary Figure S4: schematic illustration of the mechanism of the recombinant protein ephB4-Fc inhibiting osteolysis through the ephrinB2/ephB4 signaling pathway.
