# Supplementary material for: The Recombinant Protein EphB4-Fc Changes the Ti Particle-Mediated Imbalance of OPG/RANKL via EphrinB2/EphB4 Signaling Pathway and Inhibits the Release of Proinflammatory Factors In Vivo
Source: Oxid Med Cell Longev. 2020 Jun 5;2020:1404915. doi: 10.1155/2020/1404915 (PMC7294355; doi:10.1155/2020/1404915)
Supplement: Supplementary materials — Supplementary Figure S1: preoperative X-ray and postoperative prosthesis pictures. Supplementary Figure S2: immunohistochemistry of the interface membrane. Supplementary Figure S3: functionalization of osteoclast differentiation after overexpression and silencing of Raw264.7 cells was verified by western blot. Supplementary Figure S4: schematic illustration of the mechanism of the recombinant protein ephB4-Fc inhibiting osteolysis through the ephrinB2/ephB4 signaling pathway. [file 1404915.f1.pdf]

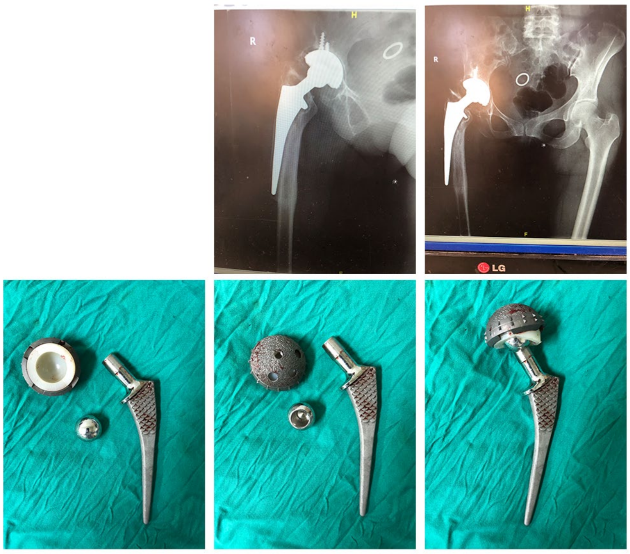

Supplementary Figure. S1.Preoperative X-ray and postoperative prosthesis pictures.

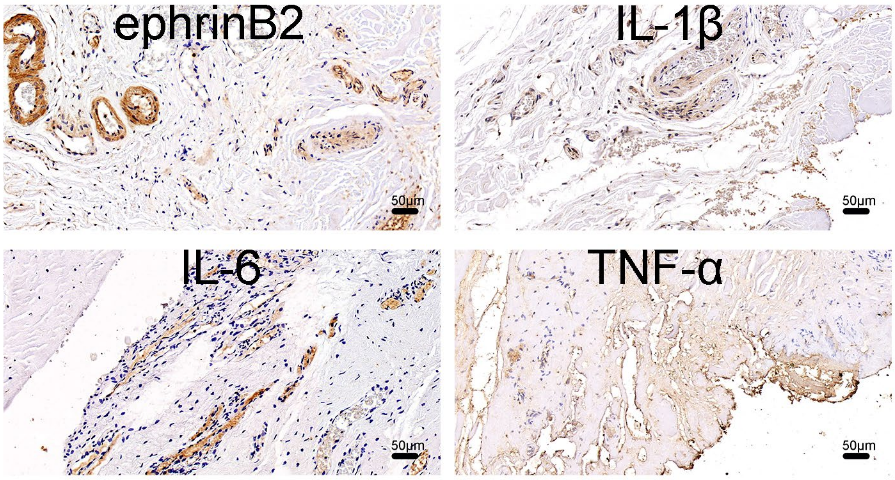

Supplementary Figure. S2.Immunohistochemistry of the interface membrane.

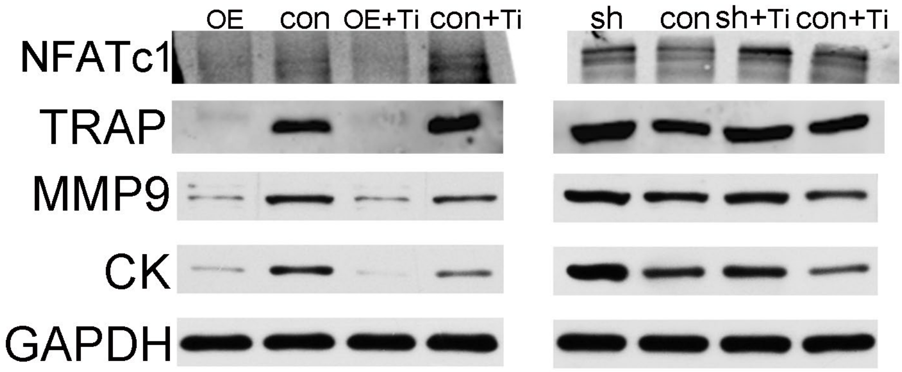

Supplementary Figure. S3.Functionalization of osteoclast differentiation after over-expression and silencing of Raw264.7 cells was verified by western blot.

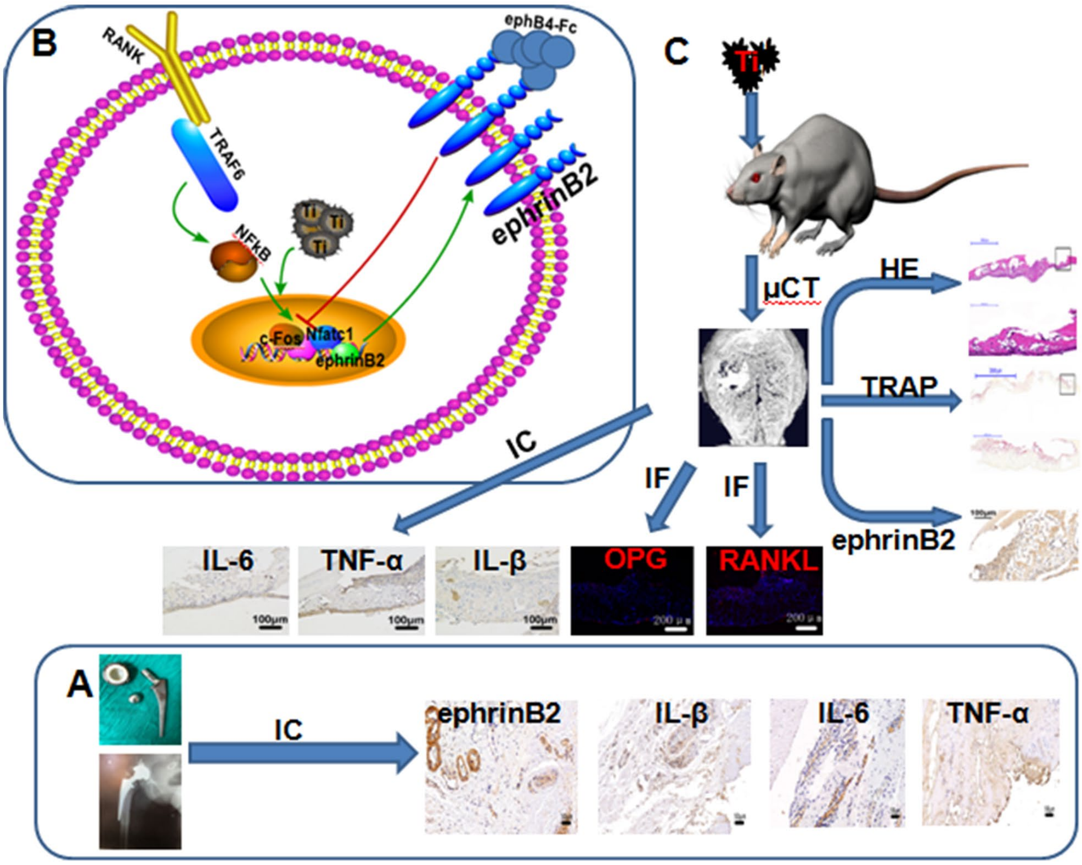

Supplementary Figure. S4. Schematic illustration of the mechanism of the recombinant protein ephB4-Fc inhibiting osteolysis through the ephrinB2 / ephB4 signaling pathway.
